# Gum-Cutting

**Published:** 1875-03

**Authors:** Charles E. Buckingham

**Affiliations:** Professor of Obstetrics in Harvard University


### GUM-CUTTING.

BY CHARLES E. BUCKINGHAM, M.D.,

*Professor of Obstetrics in Harvard University.*

Some months ago, among your "Notes and Queries," was a question about cutting gums. A week or two later, a correspondent (E. T. W.) gave a very decided answer as to the propriety and usefulness of the operation. For some time before the question was asked in the *Journal*, I had been making the same inquiry of physicians whom I met, and I was rather surprised to find among the younger practitioners so many who had never used the gum lancet, and could not imagine the case in which it would be necessary.

The let-alone system of treatment is good; in very many cases it is the best; but it is not always the best. The relief afforded by a free incision through the gum in some instances in which there was acute pain has, under my observation, been more marked than that afforded by any other operation that I ever saw. The tooth, it is true, in a very great majority of cases, finds its way through without difficulty to the child. The first indication to the mother whose child has always been nursed, and never fed, is an incisor against her nipple. In some cases where a proper plan of feeding has been followed, there is as little indication of trouble during dentition. Occasionally when the child is nursed, more frequently when it is fed, the little one apparently suffers pain in the mouth, in the head, and in the bowels, whenever a new tooth is about to make its appearance. In another class of badly-fed patients there is always loss of appetite at this time, with sleepless nights, nausea, and vomiting. In others, cough comes on which only exists then, and from which auscultation gives no explanation; and the little patient's sufferings are augmented by "hive syrup," squills, and other nauseants which give no relief, and by "Mrs. Winslow's soothing syrup," and other narcotic drugs, which stupefy but do not cure. There is still another class, consisting mainly of improperly-fed children, who have convulsions, sometimes slight, it is true, and sometimes fatal. There is no disturbance of the nervous system, so far as I know, which may not exist in the teething child, and none which may not be aggravated by improper food. Indeed, the time of dentition is the time when by far the greatest number of deaths take place among children, whether the immediate cause be in the head, the chest, or the abdomen.

There are two common notions among maiden ladies, who are often the advisers of mothers younger than themselves; these notions are the source of very great suffering to babies, and occasionally the cause of death. They are, that cutting the gum is very injurious, because "it may callous over afterwards and become so hard" that the tooth cannot get through so easily as if let alone; and secondly, "that the child may bleed to death" after the operation.

To the first of these the reply is, that no union of the gum after it has been cut will ever be any firmer than the gum itself was before it was cut. Cutting the gum may be as great a relief to an obstruction as when an incision is made over a bullet, a piece of bone, a splinter of wood, or a fragment of needle beneath the skin, and the system alone to help it to the surface. More than this, the tooth is not only below the mucous membrane of the gum, but it may be within the sac in which it was formed, and which the force of nature is trying to perforate. If this covering be once cut across, its union (if it ever unites again) will be less perfect than before, and the patient's suffering will be relieved. Suppose the gum should heal after the incision, and the child's suffering should recur; that is not a good reason for withholding relief now. And if it should suffer again, it can be relieved a second time, and even a third time. I never saw the case in which, if the gum lancet went well through, and was felt upon the surface of the tooth, there was any trouble with that particular tooth afterwards. It surely never could retract, and become deeper in the jaw than before.

The first effect of the incision is the relief of local pain by the oozing of blood. This is more particularly the case in those instances in which the gums are dry and hot, and there is no secretion from the mucous follicles nor from the salivary glands. An incision simply through the mucous membrane is followed by blood, and that by saliva in a very short time, giving great temporary relief; but if the lancet is felt to graze the tooth through the whole length of the incision, the relief is more than temporary; the immediate covering of the tooth never unites again, the growth of the tooth and elasticity of the tissue preventing that process. If the offending tooth be a molar, a crucial incision is better than a longitudinal one. The relief is often so great from gum-cutting that I have seen children who were crying with agony, before the operation, look up in my face and laugh through their tears; and I have known a child to come to me and show by unmistakable signs her remembrance of the benefit received on another occasion, by turning her head over upon my knees and pointing to the swelling above a cuspid tooth.

The second effect of gum-cutting is the relief of obstinate diarrhœa, obstinate constipation, and of all apparent signs of diseased brain, such as vomiting, stupor, convulsions, enlarged and non-contracting pupil. This relief I have seen more than once during the past year.

The second notion with which non-professional persons are possessed is the hemorrhagic. I have no doubt that in my thirty years of professional life, I have had quoted to me, on an average, more than one case a year of fatal hemorrhage from gum-cutting. But I never saw such a case, and I never spoke with a person who had seen one; it had always "been told to her" by some one else. I can imagine that it might be the possible result in one of the so-called "bleeders;" but a prick with any other instrument in any other part would in that case be likely to have the same result. The nearest approach to this condition that ever came under my observation was some ten or fifteen years ago, in the case of a young man of at least twenty-five years of age, who died of hemorrhage following the extraction of a tooth.

There is no operation, no medicine, which may not be followed by death. So sometimes neglect of medicine or neglect of an operation is followed by death. To offset any death resulting from gum-cutting (an accident which I never witnessed and never heard well authenticated), I could point to large numbers of infants whose comfort has been established, whose lives I believe to have been saved. There are many more whose comfort I believe to have been sacrificed, and whose lives I believe to have been destroyed, by the prejudice against the gum lancet.

